# A framework for the prediction of earthquake using federated learning

**DOI:** 10.7717/peerj-cs.540

**Published:** 2021-05-28

**Authors:** Rabia Tehseen, Muhammad Shoaib Farooq, Adnan Abid

**Affiliations:** Department of Computer Science, University of Management & Technology, Lahore, Punjab, Pakistan

**Keywords:** Machine learning, Federated learning, Local data model, Global data model, Earthquake early warning

## Abstract

Earthquakes are a natural phenomenon which may cause significant loss of life and infrastructure. Researchers have applied multiple artificial intelligence based techniques to predict earthquakes, but high accuracies could not be achieved due to the huge size of multidimensional data, communication delays, transmission latency, limited processing capacity and data privacy issues. Federated learning (FL) is a machine learning (ML) technique that provides an opportunity to collect and process data onsite without compromising on data privacy and preventing data transmission to the central server. The federated concept of obtaining a global data model by aggregation of local data models inherently ensures data security, data privacy, and data heterogeneity. In this article, a novel earthquake prediction framework using FL has been proposed. The proposed FL framework has given better performance over already developed ML based earthquake predicting models in terms of efficiency, reliability, and precision. We have analyzed three different local datasets to generate multiple ML based local data models. These local data models have been aggregated to generate global data model on the central FL server using FedQuake algorithm. Meta classifier has been trained at the FL server on global data model to generate more accurate earthquake predictions. We have tested the proposed framework by analyzing multidimensional seismic data within 100 km radial area from 34.708° N, 72.5478° E in Western Himalayas. The results of the proposed framework have been validated against instrumentally recorded regional seismic data of last thirty-five years, and 88.87% prediction accuracy has been recorded. These results obtained by the proposed framework can serve as a useful component in the development of earthquake early warning systems.

## Introduction

Earthquakes are catastrophic events that result in loss of life, infrastructure and have a severe impact on the economic system of a country. Numerous earthquakes occur every year around the world, killing thousands of people. Multiple computational methods have been applied for earthquake prediction, but accurate prediction of earthquakes (time and place) is still a challenging task (Jiao & Alavi, 2020). A lot of research has been conducted for earthquake prediction using different artificial intelligent (AI) approaches in the last decade (Asim et al., 2017). Tehseen, Farooq & Abid (2020a) reported that Expert systems (ES) have been widely practiced on seismic data for making earthquake predictions from 2010 to 2013. Neural network (NN) based earthquake prediction systems remained center of attention from 2013 to 2017 and were followed by an era of Machine Learning (ML) and Deep Learning (DL) based methods. Al Banna et al. (2020) has also approved that ML and DL have performed relatively better than the other computational approaches in making probabilistic forecasts about the expected magnitude of upcoming earthquakes in recent years.

Research about earthquake prediction is now significantly focused towards state-of-the-art cloud centric methodologies for efficient data communication (Lim et al., 2020). Cloud centric methodologies upload and process seismic data in a cloud server incurring high costs and unacceptable latency (Yao et al., 2019). Earthquake prediction requires multidimensional data that is naturally decentralized as data sources are located outside the cloud. Thus, the transportation of this huge seismic data to the central server also offers a big challenge (Salditch et al., 2020). Multiple streams of seismic data have been collected through sensors and aggregated at earthquake data collection centre to produce inference models. Running computational models to simulate multiple seismic data streams and transportation of these data streams as a single data model becomes computationally so hard that a research-level simulation can take weeks, even running on a supercomputer. It also evoked major issues about data privacy, data availability, data security, data protection, and data reliability. Seismic data is one of the most valuable resources, and agencies usually avoid data sharing due to data protection legislations about the sensitive nature of data (Elwood et al., 2020; Enachescu, 2007). International agencies provide open access satellite datasets with reasonable accuracy but its high cost and the issue of indigenous knowledge (IK) poses a great challenge in terms of data capturing, storage and distribution. Many societies in third-world countries lack the technicality processes of managing the IK. Due to these problems onsite regional seismic data acquisition is more popular (Zhao et al., 2020; Elwood et al., 2020).

The application of Mobile edge computing (MEC) leveraged on to bring model training closer to where data is produced. FL is an ML technique that can facilitate the earthquake prediction process by training local data models onsite and ensure data privacy at local stations. FL also applies multiple data security protocols on local data models before transmitting them to the network (McMahan & Ramage, 2017). Basically, FL is a client-server architecture that transmits local data model generated at the local station to the server machine for training global model. This feature protects the network from getting overloaded resolving latency along with other security and privacy issues (Zhao et al., 2020). Application of FL technique for earthquake prediction is also cost effective by transmitting updates only to the central server machine as compared to transmitting huge data to the server.

The main objective of this research is to ensure data availability, facilitate data collection and enforce data privacy by the application of FL technique. This research has proposed a unique framework for earthquake prediction using FL. We have collected three different datasets from Western Himalayan zone and processed them on seven different data stations to train local data model. Different ML classifiers have been trained on global data model at the central server to generate earthquake predictions. We have tested the proposed framework on instrumentally recorded dataset of Western Himalayan zone containing thirty million records from the last thirty-five years (1985–2020). The novelty of our work is that we have applied federated learning technique for earthquake prediction in Western Himalayan region by training ML based local data models onsite and transporting those models to the central server where global data model is trained by their aggregation. A stacking classifier has been trained on a global data model to generate earthquake predictions. To the best of our knowledge, this is the first research work that presents FL framework for earthquake prediction in Western Himalayan zone.

The article has been organized in the following way: “Related Work” gives a brief survey of the computational approaches used for earthquake prediction in literature. “Proposed Framework” presents the proposed FL framework for earthquake prediction. Experimental verification of the proposed framework has been performed in “Experimental Verification”. Results have been discussed in “Results and Discussion”. “Conclusion and Future Directions” concludes the article and provides future research directions.

## Related work

Researchers have been exploring the field of earthquake prediction, unfolding multiple aspects of regional or global earthquake data, performing experiments, and applying multiple computational methods to evaluate their results since long time. We have discussed some of the earthquake prediction approaches used in the literature as given below:

### Traditional artificial intelligence approaches

Artificial intelligence is the branch of science that has been used to develop intelligent machines. Artificial intelligence has emerged with many distinct approaches including expert systems (ES), machine learning (ML), deep learning (DL), neural networks (NN) and other techniques that have been applied for earthquake prediction (Lim et al., 2020; Yousefzadeh, Hosseini & Farnaghi, 2021; Aslam et al., 2021a, 2021b). Mirrashid (2014) applied Adapted Neuro Fuzzy inference system (ANFIS) to predict magnitudes of expected seismic events using Fuzzy C-means algorithm. Tehseen, Farooq & Abid (2020b) proposed Fuzzy Expert System for earthquake prediction and analyzed Himalayan data. Traditional deep neural networks involved cloud centric approach to train model in powerful cloud servers with centralized data (Raj, 2019). However, earthquake prediction process involves devices that are equipped with highly advanced sensors and computational capabilities that migrates intelligence from cloud to the edge devices. It naturally arises the need of mobile edge computing paradigm (MEC). Some streams of regional data like temperature, pressure, humidity, and animal behavior enforces MEC due to the involvement of different sensory systems in data collection. A FL based system has been applied to analyze data collected from sensory networks working in smart cities for disaster management (Jiang et al., 2020). Being wearable devices, quality of the sensor is important for reliable data collection. Quality of sensors has been monitored through a model developed by the Open Geophysical Consortium (OGC, 2020). Researchers have focused on using sensory data collected from cellular networks and social media for disaster response. Wilson et al. (2016) collected cellular network data using inertial sensors for estimation of population size and density of a specific area. Data about active mobile phone users of the region have been collected to facilitate resource allocation during disaster management focusing on post-earthquake activities. Arooj et al. (2019) proposed a framework for disaster management using smart vehicles equipped with comprehensive sensory system. Lu, Cao & Porta (2016) observed the utility of smart phones with embedded inertial sensors in the process of disaster recovery and management. Sakhardande, Hanagal & Kulkarni (2016) proposed a disaster management system by connecting sensory networks using Wi-Fi for the collection of information from sensory network and generating early warnings to multiple connected devices.

In conventional ML algorithms domain expertise were compulsory for building of an effective model. Traditional ML models have been based upon hand-fabricated feature extractors for processing raw data (Lim et al., 2020). Feature selection needed to be additionally customized and restarted for every new problem. ML is incredibly powerful in measuring environmental factors which have close relation with earthquake prediction like atmospheric temperature, atmospheric pressure, and humidity (Dikshit, Pradhan & Alamri, 2020). Lai et al. (2021) explored the impact of correlation between water temperature and water level data of Lijiang well on earthquake prediction in Yunnan, China. de Walle & Dugdale (2012) presented a model for providing the necessary services required after an earthquake to rescue missing people and providing shelter. However, network remained overloaded due to the transmission of big data to the single server.

Many researchers have applied IoT for seismic signal detection. IoT based models have been proposed in Zambrano et al. (2017), Atzori et al. (2012) for the detection of earthquakes and generating alerts to the nearby smart devices. IoT involves many wearable devices that may affect data communication due to noisy data (Pirmagomedov et al., 2018). Sarwar et al. (2019) utilized IoT to present an intelligent fire warning application. Li et al. (2017) proposed a deep convolution computation model for learning features of big data using IoT. Ray, Mukherjee & Shu (2017) emphasized on the importance of IoT in efficient resource management, defining recovery policy and fast communication for disaster management. Lu et al. (2020) incorporated privacy-protected FL technique to address data sharing problem by communicating data model instead of revealing actual data using blockchain mechanism.

### Federated learning (FL)

Regional seismic data collection involves data privacy, data availability and data communication concerns imposed by data protection legislations (Enachescu, 2007; Elwood et al., 2020). To mitigate these challenges the concept of FL has been proposed (Aledhari et al., 2020). FL alleviates the privacy concerns by allowing the users to collaboratively train a shared model while keeping personal data safe on edge devices (Mothukuri et al., 2020).

There are two main elements of the FL system including the owner of the data (edge devices) and owner of the model (FL server). If *N* = {1,2,3…*N*} is a set of data owners, each with its own data set D_i_ then every owner N_i_ will use its dataset Di to train local data model w_i_ and transmit only local model parameters to FL server. It shows that data itself do not travel along the network rather a small sized local data model is transferred to the server for training global data model. FL server aggregates all received local data models to generate a single global model. It is different from traditional centralized training in which data from each source is aggregated first to centrally train the model.

FL has vast application in many fields and is getting attention due to the advantage of 4G technology. Arooj et al. (2020) proposed a route selection model using 4G internet technology in which smart vehicles are connected to the central system that can automatically select and alter their route depending on the type of signal they have received from the central system. Some researchers have integrated FL with block chain technology (Kim & Hong, 2019; Farooq, Khan & Abid, 2020; Majeed & Hong, 2019; Huang et al., 2020; Weng et al., 2019). The FL system has been applied on synchronous training of algorithms (Lim et al., 2019), vehicle-to-vehicle communications (Samarakoon et al., 2018), medical applications (Brisimi et al., 2018), and for earthquake early warning system using smart phones (Kong et al., 2016). The FL model has been used to transmit data recorded by seismographs installed nationwide on the centralized server for the prediction of earthquake (Minson et al., 2018; Sims, 2015).

The above discussion revealed that many efforts have been carried out to predict earthquakes using multiple computational approaches, yet no adequate procedure for the prediction of earthquakes (place and time) yet exists. In this article, we have applied federated learning technique for earthquake prediction. ML based local data models have been trained by onsite data collection and aggregated at the central server to generate global data model. Meta classifier has been trained on global data model for more accurate prediction of earthquakes. To the best of our knowledge, none of the studies has presented FL framework for earthquake prediction in Western Himalayan zone.

### Proposed framework

**Figure 1:**
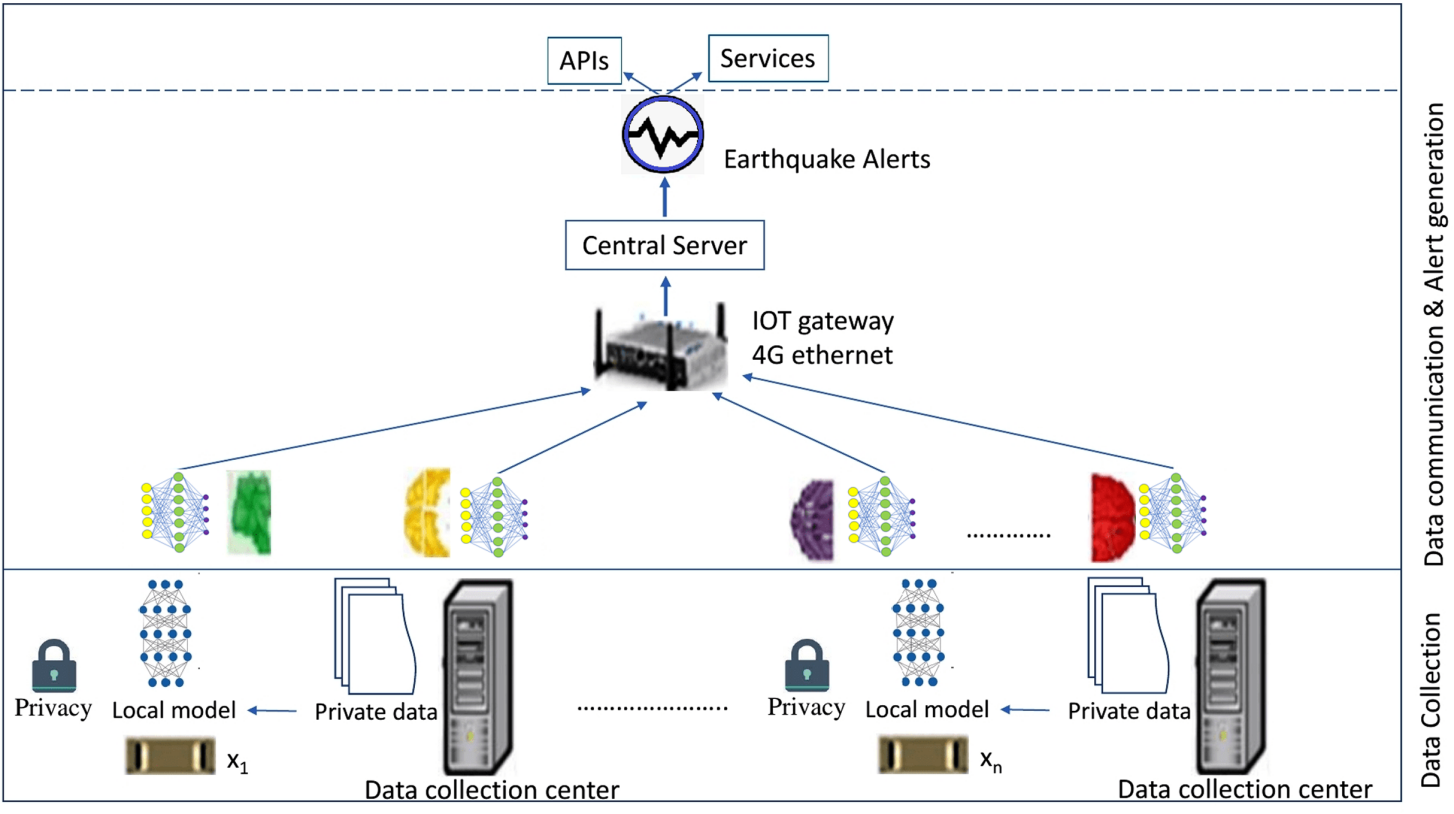
FL framework for earthquake prediction.

In this section, the framework for earthquake prediction using FL has been proposed. In this framework many rules, functions that are defined according to the expert judgments and mathematical formulas are applied on local stations for extracting significant parameters from local datasets. Different ML techniques have been used to train local data models by processing local datasets onsite. Local data models have been transmitted through network to the FL server where global data model is trained by aggregating local models using FedQuake algorithm. Then, meta classifier has been trained on global data model to generate earthquake predictions with more accuracy. The proposed FL framework has been presented in Fig. 1. It comprises of two basic layers namely data collection layer, data communication and alert generation layer. The architecture and layer-wise functions of proposed framework is described below:

### Architecture

The basic structure of the proposed framework and federated learning mechanism has been presented below:

#### Structure

Proposed framework has been implemented using TensorFlow. To perform FL in the proposed framework, all client computers run a software package that contains a Delay Tolerant Networking (DTN) layer, a local controller to respond to requests, and TensorFlow federated (TFF) layer. TFF platform consists of two layers namely FL and federated core (FC). We have used FC to formulate the foundation of FL in the proposed framework. It has provided us a system of low-level interfaces for writing federated algorithms in combination with distributed communication operations in strongly typed functional programming environments. With FC, new data type can be expressed, specifying its underlying data and where that data lives on the distributed clients. In the proposed framework, FC made it possible to combines TensorFlow code with distributed communication that operates over a set of client devices in the system for broadcasting client models to connected devices. In our work, tff.Client represents all the clients and tff.Server is a single physical device representing server machine. tf.contrib.distribute library enabled clients to use existing models and training code for distributed training. TFF treats federated data as First-Class citizen allowing it to be in the form of parameters or result of the functions.

#### Learning mechanism

To perform ML in the proposed framework, FL server uses federated data for model training and evaluation that is like device own data. Main training loop takes place on decentralized data. The FL server performs actions like ML workflow by starting and stopping tasks, calculating learning rate, and monitoring their performance during the training process. If the model performs well on the decentralized dataset, then the FL server has the good release candidate. Server will validate this release candidate using evaluation techniques. Initial model as set by the FL server is send to the client. The client completes the local updating of the model using their own data. Only these updates are then sent to the server for the aggregation. Other devices are also participating in this round performing their own local updates to the model. Some of the clients might drop out before recording their updates. The server will aggregate all updates into a new model by averaging all model updates. Updates are federal and will be discarded after use. FL server keeps on monitoring the performance of federated training through matrix aggregated along with the model. Training rounds will continue only if the model performance is satisfying the threshold. A different subset of devices is chosen by the server in next round and given the new model parameters. This is an additive process and will continue to many training rounds. Federated averaging algorithm is applied for computing data weighted average from many steps of gradient descend on the device. The FL server is responsible for collecting device reports and draw rounds. It combines the reports of multiple clients using federated aggregation computation. FL server will repeat these steps and can select different set of devices in every round as some devices may not be available at the time of federated computation or some may be dropped out due to certain reason. After coordination by the server, all devices participating in every round agree to share their weight to calculate the sum by exchanging zero sum pairs. Clients calculate their weight and update the model received from server. Devices clip their updates if they are too long as model updates are limited to a specific size. Then the server will add noise of the same magnitude to normalize maximum size of updates that any one client can send while combining the device updates of that round. These values are then aggregated using an aggregation operator, in this case it is a federated mean which includes a protocol for computing the average value over the participating devices. Federated averaging algorithm (FedQuake) presented in this article is used for the calculation of federated mean.

### Layer-wise functionality

The proposed framework consists of two layers. Functions performed in every layer have been presented below:

#### Data collection (Layer 1)

Earthquake prediction requires a huge amount of heterogeneous data. In the proposed framework, seismic dataset, climate dataset and reservoir dataset from Western Himalayan zone have been recorded through different networks in Fig. 1. Statistics of collected data has been presented in Table 1. Dataset have been locally processed to train local data models onsite. Multiple FL parameters like local batch size (b) used at each learning iteration, local learning rate (η), number of training (e), termination criteria and time (t) required in every training iteration has been calculated in two folds. On device training is referred as first part. In T = 0 time, the device receives a trained model named w_0_. With this model, the server also sends the mini-batch size (b), learning rate (η), number of trainings (e) and other required parameters. The model is trained on the device when data is collected in a threshold amount. It can be shown as w_1_ = model (x, y, b, e, η). Here, w_1_ is the new weight matrix calculated by the model and ‘x’, ‘y’ are input and destination output on the local devices respectively. In the second part, the server collects locally produced trained weights from the devices. Now, the weight matrix collected from ‘n’ devices represented as w_1n_ takes place as given in Eq. (1).

**Table 1:** Statistics of the collected data.

Stations	Local data models	Data parameters used to train the model										
		Seismic data				Climate data			Reservoir data			
		Maximum magnitude	Seismicity level	Depth	Magnitude ratio	Temperature	Pressure	Rainfall	Water level	Water state	Natural contents	Saturation format
A	x1	✓	✓	✓	✓	✓	✓	✓	✓	✓	✓	✓
B	x2	✓	✓	✓	✓	✓	✓	✓				
C	x3	✓	✓	✓	✓	✓	✓	✓	✓	✓	✓	✓
D	x4	✓	✓	✓	✓	✓	✓	✓	✓	✓	✓	✓
E	x5	✓	✓	✓	✓	✓	✓	✓	✓	✓	✓	✓
F	x6	✓	✓	✓	✓	✓	✓	✓				
G	x7	✓	✓	✓	✓	✓	✓	✓	✓	✓	✓	✓

Total number of data points in all devices = N

Number of local data points used to obtain w_1n_ from Z devices = n

(1) $$w_{1n}=\frac{\left(n\times w\right)}{N}$$

It considers a small portion of clients (Z) in each round to update overall weights. Here n_z_ is the number of randomly selected clients as given in Eq. (2).

(2) $$n_{z}=\max(Z*N,1)$$

The global and local code of the federated averaging algorithm (FedQuake) has been presented below:

**Algorithm 1:** FedQuake

1: **procedure** ServerUpdate
2: Initialize w_0_
3: **for** each round t = 1,2… **do**
4: *m←max(C.K,1)*
5: *S*_*t*_*←random set of m clients*
6: ***for*** *each client k ⊆ S*_*t*_ *in parallel* ***do***
7: $w_{t+1}^{k}\leftarrow ClientUpdate\left(k,w_{t}\right)$
8: ***end for***
9: ***// privacy selection***
10: $w_{t+1}^{k}\leftarrow \sum_{k=1}^{K}\frac{n_{k}}{N}w_{t+1}^{k}$
11: **end for**
12: **end procedure**
13: **procedure** Client Update (*k*, ω)
14: **// run on client k**
15: β←Split data m_k_ into batches of size β
16: **for** each local epochs i = 1 to ε … do
17: **for** batch b ∈ β **do**
18: w←w−∇(w)
19: **end for**
20: **end for**
21: *return w to server*
22: **end procedure**

In the FedQuake algorithm, the central server is started with the weight w_0_. Once started, the server communicates simultaneously with local devices. At time t, server shares central model w_t−1_ with a sub-set of the clients S_t_ randomly selected from the user pool K with a participation rate C. Each client k ∈ S_t_ performs one or more training rounds through local data with a local learning rate η. S_t_ clients send model updates (w_t_, k where k ∈ S parameter) back to server after completing local training. The server calculates an average model based on updates received from local models. Denoting T_local_ as the time to computing one local round then the computation time for one global round becomes λT_g_ where λ represents communication delay. The wall clock time of one global round using FL is defined as (3)

(3) $$T_{g}=T_{local}+\lambda T_{global}$$

#### Data communication and alert generation (Layer 2)

FedQuake algorithm has been used for selecting subset of clients to participate in a particular training round. TFF defines federated computation parameters to run in a decentralized setting. They also include a local-machine runtime to simulate the computation being executed across a set of clients holding data, their local contribution, and the centralized coordinator module aggregating all the contributions. During task initiation process, a set of specifications for the FL task such as model architecture, data type, and expected prediction labels are broadcast to all other clients in the network. The DTN layer ensures that each client will receive the specifications. After receiving these specifications, each client checks whether it has the data that can be useful for the FL task and whether it has enough computational resource to participate in the task. If both are true, the client will send a confirmation to the initiator of the task. FL starts when a waiting time has elapsed after task initiation that is determined by the task initiator and is dependent upon minimum number of participating clients. After the model is trained, it can either be used locally or re-distributed to other participating clients if they require the model. In Fig. 1, Layer-2 is responsible for data communication between FL-clients and FL-central server. It establishes two-way communication between federated clients and federated server machine by receiving local data models from data collection layer and transporting them to the server for processing. Local data models are received by the server and further processed to generate global data model. Multiple ML techniques are applied to trained local data model and meta classifier is trained on global data model to make accurate predictions. Earthquake predictions generated from the server are forwarded to the concerned departments for alert generation using Global system for mobile(GSM). GSM is a mobile communication modem developed at Bell laboratories in 1970. GSM is widely used, open digital cellular communication technology used to transmit data at a data rate of 64 kbps to 120 Mbps. Through this layer, predictions made by proposed framework are transmitted to the concerned departments and agencies responsible for hazard mitigation and response. These agencies also use GSM module to issue earthquake early warning alerts for all connected smart devices.

## Experimental verification

In this research, three different streams of data have been collected and processed using FL approach. For experimental verification, we have recorded climate dataset, seismic dataset and reservoir dataset from seven different stations (A, B, C, D, E, F and G) located in 100 km radial area from point 34.708° N, 72.5478° E. To perform detailed experiment, regional instrumental data from 1985 to 2020 has been collected and divided into 80% training dataset and 20% testing dataset. Experiment has been performed in two different dimensions. In first dimension, we have generated basic learning model (BLM) using relational data base system. Model has been trained on the dataset containing records from 1985 to 2015. Multivariate rules and formulas have been developed and applied to determine relationships among different data parameters for predicting the magnitude of the earthquakes encountered during 1985 till 2015 in the region. BLM has been tested on the dataset containing records from 2016 to 2020.

In second dimension, the proposed FL framework has been implemented using TensorFlow platform. Working of the client is expressed in following five steps. In the first step, dataset have been collected onsite to generate federated learning model (FLM). In FLM, seven different stations are recording data with respect to climate, seismicity, and reservoir. In the second step, some pre-processing of data is performed at edge that include normalization of data and importing multiple libraries of TensorFlow. In third step, the architecture of proposed framework has been defined that includes the type of ML classifier, batch size (b), learning rate (η) and number of trainings (e). Seven ML based local data models have been developed and their weights are updated to the central server (CS). These local data models are collected using FedQuake algorithm at the CS which is responsible to update the state of the global variables presented in Table 2 in the TFF script. As we are using one CS and seven clients for three datasets, the script will be launched eight times once at every terminal and on the server. In fourth step, data clusters have been defined using tf.train.ClusterSpec. For initialization of data clusters, a dictionary with CS and client keys is received as initialization parameters. During an iteration, script running on CS will be continuously at ‘listen and wait’ state for ‘read or write’ operation from clients. After a specific time, the script running on CS will be blocked to complete initialization of parameters and the action of client nodes will start. Working of federated clients is expressed in Table 3.

**Table 2:** State of FL variables.

Flag title	Label	Description
Job Name	CS	A string denoting central server or client
Task Index	0 to 7	An integer to distinguish CS and client
CS Host	0	A string containing address of CS
Client Host	Client	A string containing address of client

**Table 3:** Working of Federated clients.

Steps	Description
**Step 1**	Clients train on a batch locally
**Step 2**	In every iteration, client will update its weights as if isolated training is being performed
**Step 3**	All clients would transmit weights according to their configuration
**Step 4**	Federated averaging algorithm will collect local data models and calculate average and update the shared model to CS
**Step 5**	On receiving the updated average. CS sends a message to rest of the clients to pull the latest model before training on future batches

In the final step of client’s working, tf.device creates a device type object and replica.device.setter takes care of the synchronization between the clients. Every client will receive data model from the server. For generation of global data model, every client will add its updates to the received model and model will be passed back to the central server. Server asks all the clients to pull the updated model for further processing. This process continues until a good performance matrix is achieved. At the same time, regular interval check points are also loaded and maintained to evaluate the model as training model ceases and does not exist as soon as session is closed. After successfully generating global data model, meta classifier is trained on it to make earthquake predictions.

**Figure 2:**
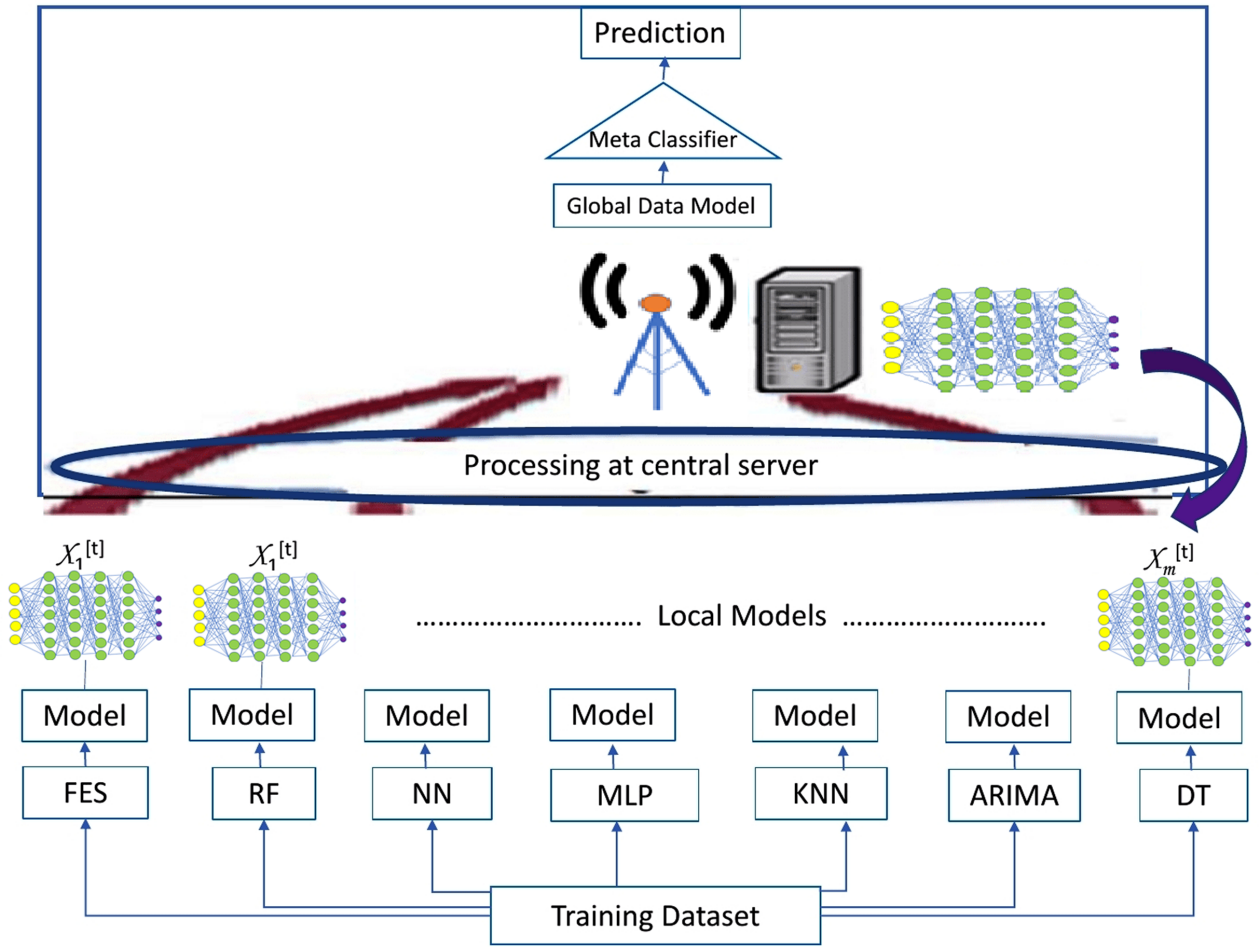
Ensembling architecture.

Multiple predictive modeling methods of statistics, data mining and ML are applied to train the classifiers at clients for generation of local data models. Different ML classifiers including decision tree (DT), neural network (NN), multilayer perceptron (MLP), k-nearest neighbor (KNN) and random forest (RF) have been trained on clients as presented in Fig. 2. Fuzzy Expert system (FES) as a traditional benchmark [12] and Autoregressive integrated moving average (ARIMA) method of time series has also been applied for the generation of local data model at clients. Performance of these classifiers and methods have been compared in terms of prediction accuracy, precision, recall, f1 score and rate of data loss using Eqs. (4)– (6).

(4) $$Precision=\frac{TP}{TP+FP}$$

(5) $$Recall=\frac{TP}{TP+FN}$$

(6) $$F1\;\left(SCORE\right)=2\times \frac{Precision\times Recall}{Precision+Recall}$$

Various parameters (P) are tuned for comparative analysis of all methods along with their values (V) are described in the Table 4.

**Table 4:** Parameters tuned for analysis of classifiers.

MLP	P	Activation	Early-stopping	Hidden layersize	Learning rate	Learning rate init	Solver	Toll
	V	ReLU	True	(5,5,5)	Adaptive	0.001	Sgd	0.0001
KNN	P	N_neighbour		Metric		n-jobs		Weights
	V	12		Euclidean		None		Uniform
DT	P	Max_depth		Criteria		Min_sample_leaf		
	V	100		Entropy		5		
RF	P	Max_depth		Criteria		Min_sample_leaf		n_estimators
	V	100		Entropy		1		200
NN	P	Data division		Training		Performance		Derivative
	V	Random		Levenberg_Marquadt		Mean squared error		Default
FES	P	MFs	Nodes	Linear parameters		Nonlinear parameters		Total Parameters
	V	[3,3]	870	2,132		60		2,192
ARIMA	P	*p*-value	d-value	q-value		Covariance type		Performance
	V	5	1	0		Opaque		Mean squared error

## Results and discussion

The process of FL presented in this research shows that every participating node initializes the training independently as there is no other participant in the network. Learning parameters contains the declaration of local epochs used by each participant for training. The local updates are computed and sent to the central server after specific number of epochs. The central server receives updates from every participating node, calculates their averages and aggregate them to generate the global model. The participants conduct next round of training process based on this updated model received from central server. This mechanism keeps on repeating until the given communication rounds are complete or the desired convergence level is achieved.

Results of the experiment obtained from the application of seven different ML methods have been compared in this section. We have processed three different datasets with 3,000,000 total records divided into 5 folds with batch size of 500 records collected from seven different points in Western Himalayas. Performance comparison of multiple methods has been presented in the Table 5.

**Table 5:** Performance comparison.

Parameters	Methods							
	BLM	FLM						
		DT	NN	MLP	KNN	RF	FES	ARIMA
Training time (seconds)	31	10.7	17.3	25	17.78	16	28	10.5
Accuracy	90.7	88.56	87.12	60.68	72.83	75.57	47.58	89.54
Precision	92.55	91.62	86.97	63.83	72.04	85.81	41.07	91.79
Recall	91.84	90.73	81.57	51.49	70.42	79.18	40.71	90.24
F1-Score	23.05	22.79	21.05	14.25	17.81	20.59	10.22	22.75
Data Loss	0.332	0.011	0.018	0.613	0.581	0.019	0.831	0.013

To observe the behavior of machine learning models, training process has been started with a small-sized dataset containing 10,000 records. Then in next iteration 90,000 more records were added and model was trained with 100,000 records. In third iteration, full dataset containing 600,000 records has been used for training the model. To maintain consistency among iterations 600,000 more records are added in every iteration to train the model. Some ML classifiers have been trained on local data at clients are presented below:

**Multilayer perceptron** **(MLP)** belongs to a class of feedforward artificial neural network (ANN) composed of multiple layers of perceptron with threshold activation. An MLP consists of an input layer, a hidden layer and an output layer. MLP exercises backpropagation supervised learning technique for training. In the proposed framework, MLP classifier has been trained on local data at client to process regional seismic data that is not linearly separable.

***k-nearest neighbor (k*-NN)** is one of the ML classifiers used for classification and regression. In the proposed framework, k-NN works by calculating the distance between neighbors for classification. The neighbors are selected from a pool of objects with known class or the object property value. Training data is normalized to improve accuracy of results as regional seismic data has been recorded in different scales. It assigns weight to the contributions of the neighbors and in result the nearer neighbors contribute more to the average than the more distant ones. In the proposed FL based framework for earthquake prediction, k-NN has been applied on local dataset at client due to its sensitivity to the internal structure of multi scaled regional dataset.

**Decision Tree (DT)** is a ML classifier used to build a tree by setting different conditions on its branches. It consists of a starting point called a root node, a point where splitting takes place called internal nodes and terminal nodes called leaves. DT classifier has been trained for earthquake prediction in the proposed framework due to its ability to handle complex data according to the expert’s description about a situation incorporated as a set of rules.

**Random Forest (RF)** is a ML technique that constructs multiple independent DTs under variable conditions. In this technique data arriving at root node is forwarded to all sub-trees for to predict class labels. At the end, voting is performed and the class in majority is assigned to that data. RF classifier has been trained in the proposed framework due to its ability to process big data and generate reasonable predictions with very little configuration requirements.

**Figure 3:**
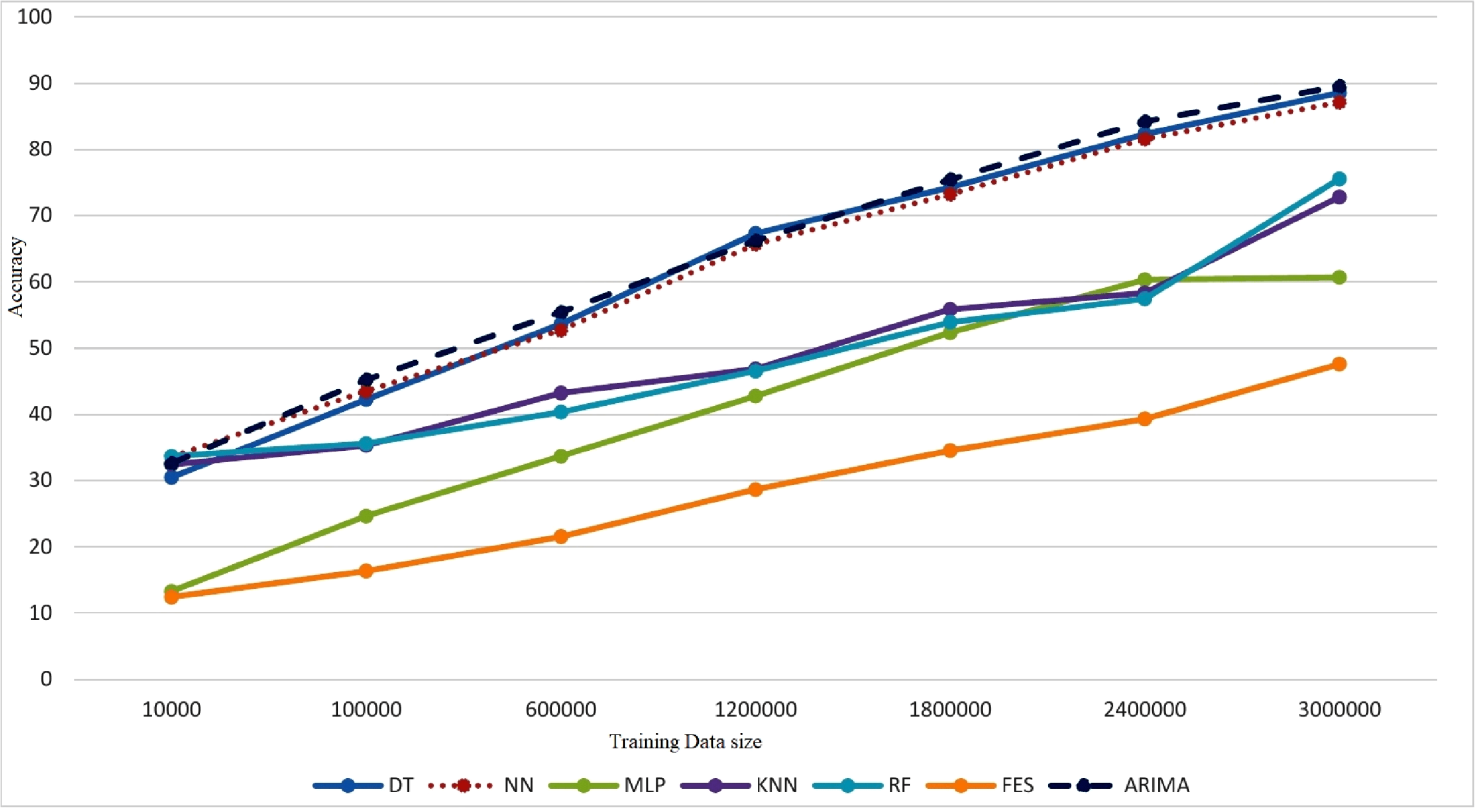
Model performance evaluation.

Performance of classifiers has been evaluated in terms of training time, accuracy, precision, and data loss by training meta classifier. Figure 3 shows that after 100,000 records KNN and RF has very low increase in accuracy indicating very moderate improvement. DT, NN and ARIMA presented a bit lower accuracy with 100,000 records of training data but as more records were added a continuous increase in accuracy has been observed. MLP and FES has shown very low accuracy with initial 100,000 records and minor improvement in accuracy has been observed when 600,000 records were added.

In case of basic learning model (BLM), centralized dataset has been used to train the model. Training of the model took place on the same node without involving any communication delay or overhead cost. It has taken more time while training as computing capacity of the single node is limited. Table 5 presents training time, accuracy obtained and emerging data loss of every model.

FLM has overcome the latency issue as clients are not generating the gradients rather small sized local models are being generated by clients and transmitted to CS. Time taken and accuracy achieved by applying FLM has been presented in the Table 5. It has been observed that less time is taken while training and transmitting the model as huge sized data is not travelling along the network. More accuracy has been obtained by the application of FL mechanism. Data loss calculated is also minimum as FL ensures data privacy and data security by the development of local data models onsite. It can be observed that the earthquake prediction model generated using FL approach is most efficient in terms of time and with minimum data loss than BLM. Accuracy of BLM with centralized data is a bit more than FLM but there is a tradeoff when data privacy, data security and data availability is more important as in the case of earthquake prediction. FL mechanism facilitated the earthquake prediction process by secured and privacy protected data collection. However, there are certain limitations associated with FL based earthquake predicting system.

During learning process, nodes frequently communicate with each other and with the FL server. Technically, FL mechanism requires sufficient local computing power, memory, and high bandwidth connections for exchanging parameters of local machine learning model. Moreover, it also needs an efficient algorithm that may reduce the number of communication rounds and send model updates in iterations as a part of training process. The provision of FL to allow clients to join and leave at any time makes it susceptible to cyber-attacks as an opponent can join the system using different aliases. FL mechanism must allow only a small fraction of the devices to be active at once and it must handle the dropped devices. The hardware variability of participating devices may also affect storage, computational, and communication capabilities of the system. FL protects on device data by sharing model updates only on the network. But transmission of model updates during the training process can still reveal sensitive information to any third party or to the server. Moreover, heterogeneity of local datasets in terms of size, population, variance with time, interoperability among nodes and node failure are the major limitation for earthquake predicting FL based systems.

**Figure 4:**
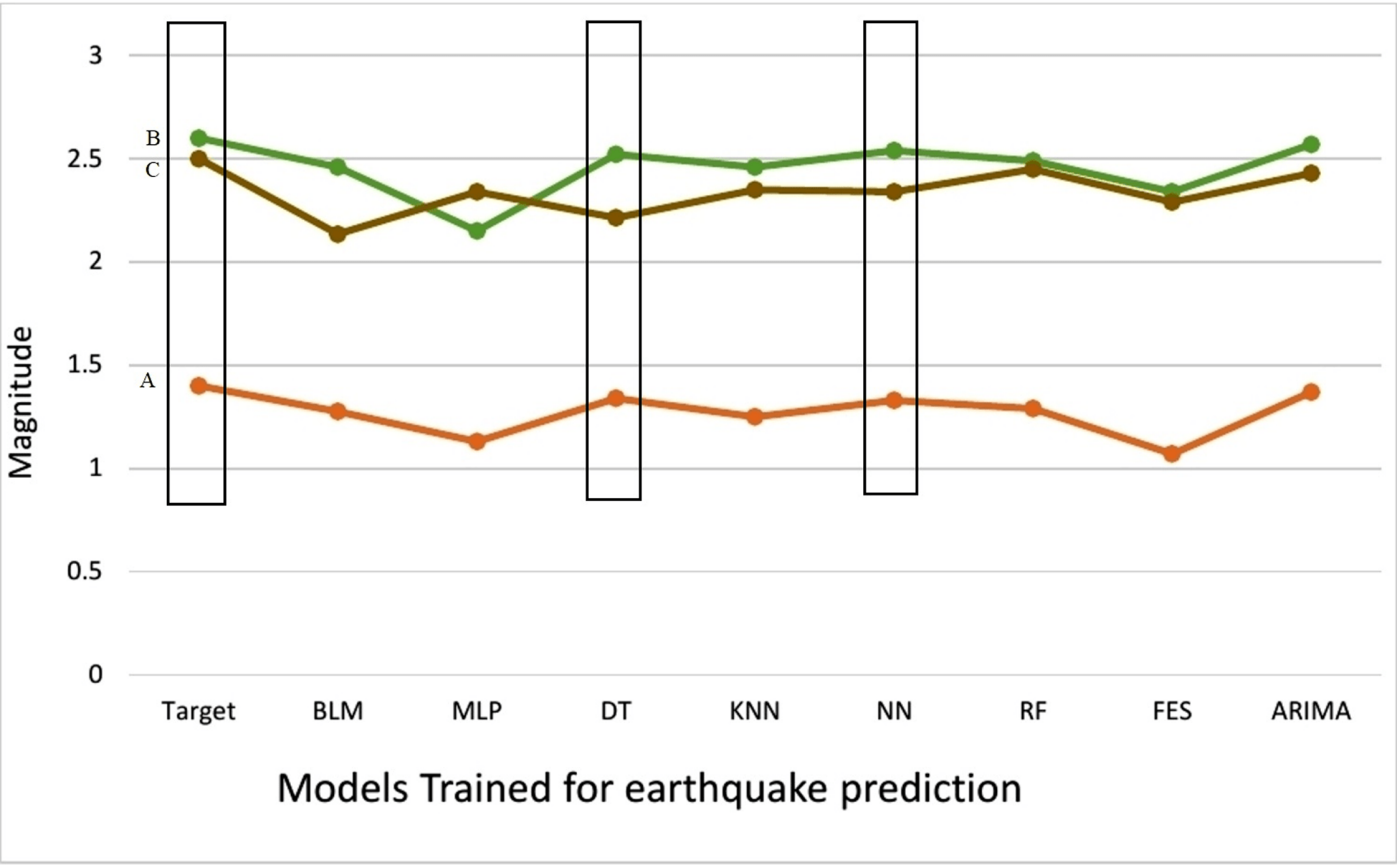
Comparison of predicted earthquakes against target.

We have compared the predictions made by different machine learning models with actual earthquakes that have encountered in the region presented in Table 6. We have set the threshold value of magnitude to 5.0 to show that FLM is capable to predict earthquakes that are above threshold value. It can be clearly observed from Fig. 4 that DT, NN and ARIMA have been most effective in earthquake prediction research. Meta classifier has been trained on global data model that compared the prediction accuracy of multiple ML models used to generate global data model and predicted earthquakes encountered in the selected region more accurately.

**Table 6:** Comparison of results with actual earthquakes at threshold magnitude 5.0.

Station	Date	Target	BL M	ML classifiers						
				MLP	DT	KNN	NN	RF	FES	ARIMA
Quetta (A)	29-10-2008	1.4	1.276	1.13	1.34	1.25	1.33	1.29	1.07	1.37
Balakot (B)	8-10-2005	2.6	2.459	2.15	2.521	2.46	2.54	2.49	2.34	2.57
Islamabad (C )	25-07-2015	2.5	2.135	2.34	2.214	2.35	2.34	2.45	2.29	2.43

## Conclusion and Future Directions

In this article, FL has been applied for earthquake prediction that enabled distributed clients to collaboratively learn a shared prediction model while keeping all the training data at its origination place. It decouples the ability to do ML from the need of storing big data in the cloud. FL leveraged model training to the edge devices where data is actually residing. The proposed FL based framework, data not only remained decentralized on client networks but some preprocessing has also been performed onsite for the extraction of significant parameters. Multiple ML techniques have been applied on local dataset collected from different stations to generate local data model comprising of only those parameters that are significant in terms of earthquake prediction. These local data models have been sent to the FL server for training of the global model. Meta classifier has been trained on global data model to improve accuracy in earthquake prediction. In the proposed architecture, data remains secure at its original place as it does not travel along the network. This feature will be beneficial for future researchers in developing secured and privacy protected systems for earthquake prediction to process data onsite without involvement of cloud. Although, most of the network security issues do not arise, but we strongly argue that data security and privacy are not binary and can present a range of threats offering new challenges for future researchers. Efficiency in terms of speed and accuracy has also been gained in the proposed architecture as big data are not moving along the network, but a small sized data model extracted from this big data has been sent on the network for further processing. However, the proposed system imposes efficiency requirements on the algorithms used to extract data model from client machines and needs to be improved in future. We have tested the proposed framework using different datasets from Western Himalayans. However, in future work the generalization ability can upgrade proposed framework to process dataset from any other region. Moreover, accuracy in earthquake prediction can be enhanced in future work by integrating federated learning with time series methods or establishing a block chain between research centers for big data and data sites. In the proposed framework, creation of large models and sending full model updates can create a bottleneck due to asymmetric internet connection speeds. New research about modern methods that might be helpful in reducing the uplink communication costs need to be investigated in future. Methods for the scalability of large sized models generated from onsite seismic data also need to be explored in future. Moreover, methods to determine how much communication is necessary in FL framework, also need to be discovered in future.

## Supplemental Information

10.7717/peerj-cs.540/supp-1 Supplemental Information 1 Code for earthquake prediction.

10.7717/peerj-cs.540/supp-2 Supplemental Information 2 Multidimensional data for earthquake prediction. Data collected from the Western Himalayas about atmospheric temperature, atmospheric pressure, reservoir level and seismicity used for the prediction of earthquakes in the region.

## Additional Information and Declarations

### Data Availability

The following information was supplied regarding data availability: The codes and data are available in the Supplemental Files.
